# Combination immunotherapy with α-CTLA-4 and α-PD-L1 antibody blockade prevents immune escape and leads to complete control of metastatic osteosarcoma

**DOI:** 10.1186/s40425-015-0067-z

**Published:** 2015-05-19

**Authors:** Danielle M Lussier, John L Johnson, Pooja Hingorani, Joseph N Blattman

**Affiliations:** Molecular and Cellular Biology Graduate Program, Arizona State University, Tempe, AZ 85281 USA; Center for Infectious Diseases and Vaccinology, BioDesign Institute, Arizona State University, 727 E. Tyler St., Tempe, AZ 85287 USA; Center for Cancer and Blood Disorders, Phoenix Children’s Hospital, Phoenix, AZ 85016 USA

## Abstract

**Background:**

Osteosarcoma is one of the most common bone cancers in children. Most patients with metastatic osteosarcoma die of pulmonary disease and limited curative therapeutic options exist for such patients. We have previously shown that PD-1 limits the efficacy of CTL to mediate immune control of metastatic osteosarcoma in the K7M2 mouse model of pulmonary metastatic disease and that blockade of PD-1/PD-L1 interactions can partially improve survival outcomes by enhancing the function of osteosarcoma-specific CTL. However, PD-1/PD-L1 blockade-treated mice eventually succumb to disease due to selection of PD-L1 mAb-resistant tumor cells. We investigated the mechanism of tumor cell resistance after blockade, and additional combinational therapies to combat resistance.

**Methods:**

We used an implantable model of metastatic osteosarcoma, and evaluated survival using a Log-rank test. Cellular analysis of the tumor was done post-mortem with flow cytometry staining, and evaluated using a T-test to compare treatment groups.

**Results:**

We show here that T cells infiltrating PD-L1 antibody-resistant tumors upregulate additional inhibitory receptors, notably CTLA-4, which impair their ability to mediate tumor rejection. Based on these results we have tested combination immunotherapy with α-CTLA-4 and α-PD-L1 antibody blockade in the K7M2 mouse model of metastatic osteosarcoma and show that this results in complete control of tumors in a majority of mice as well as immunity to further tumor inoculation.

**Conclusions:**

Thus, combinational immunotherapy approaches to block additional inhibitory pathways in patients with metastatic osteosarcoma may provide new strategies to enhance tumor clearance and resistance to disease.

**Electronic supplementary material:**

The online version of this article (doi:10.1186/s40425-015-0067-z) contains supplementary material, which is available to authorized users.

## Background

The effectiveness of conventional therapies for metastatic osteosarcoma has remained unchanged over the last thirty years, with a dismal five-year survival rate of less than 20% [1-5]. We have recently shown that metastatic osteosarcoma tumors, but not primary tumors, become resistant to CD8 T cell-mediated control due to upregulation of inhibitory receptors that limit T cell function [6]*.* Specifically, in the K7M2 mouse model of metastatic osteosarcoma, expression of programmed death receptor-1 (PD-1) and interaction with its ligand PD-L1 on tumor cells, tolerizes tumor-reactive T cells inhibiting their cytokine production and cytotoxic function towards the tumor. Moreover, both our lab and others have shown that PD-L1 is expressed on human metastatic osteosarcoma tissue, while CTL infiltrating human metastatic osteosarcomas are positive for PD-1 [7]. Therefore, immunotherapy, specifically the use of antibody blockade of such inhibitory proteins, may be an effective option for treating metastatic osteosarcoma by re-invigorating tumor-reactive T cells that can mediate tumor eradication. In support of this idea, our previous data shows that α-PD-L1 antibody blockade partially improves T cell function *in vitro* and *in vivo* and results in longer host survival with fewer pulmonary metastases during disease progression [6]. Unfortunately, metastatic osteosarcoma tumor-bearing mice treated with α-PD-L1 mAb ultimately succumb to pulmonary disease, with larger overall metastases that become resistant to PD-L1 antibody therapy. Therefore, combinational immunotherapy, by blockade of alternative inhibitory receptor pathways on T cells or accessory regulatory cells may lead to more efficient restoration of T cell function and improve control of metastatic osteosarcoma tumors.

In other experimental and clinical systems, combinational immunotherapies have shown synergistic effects on the ability of T cells to mediate clearance of tumors, and in some cases have led to complete control of tumor growth. Curran et al. combined α-CTLA-4 and α-PD-L1 mAb treatment of an implantable model of B16 melanoma and observed more than a 2-fold increase in tumor rejection compared to α-CTLA-4 mAb alone treated groups [8]. Combinational immunotherapy using antibodies to CTLA-4, PD-1/PD-L1, with small molecule inhibitors of indolamine deoxygenase (IDO), have also been shown to lead to improved tumor control and increased IL-2 production by tumor infiltrating lymphocytes (TILs) in an implantable melanoma setting [8,9]. In humans, ipilimumab (α-CTLA-4) and nivolumab (α-PD-1) mAb combinational blockade has shown promise in clinical trials against advanced melanoma with 40-50% of patients achieving objective responses and a reduction in tumor burden [10]. However, an extremely small study, looking at 4 synovial sarcoma patients treated with varying doses of ipilimumab showed no responses [11]. However disease was very advanced, and these blockade inhibitors may be best for treating patients with early metastatic disease, as cells that newly escape into the periphery may co-opt the use of inhibitory receptors to suppress T cell mediated killing of malignant cells [12]. These immune checkpoint blockade strategies also appear to enhance clearance of tumors when used in combination with chemotherapy, including during treatment of pancreatic cancer in murine models [13].

In our current study, we focus on the mechanism of resistance of K7M2 metastatic osteosarcoma cells after α-PD-L1 blockade. Attempting to understand the mechanism of resistance leads us to more beneficial combinational approaches against metastatic osteosarcoma. We will investigate if any combinational approaches overcome mechanism of resistance, and provide either significant decreases in tumor growth or complete protection. Evidence from our model will provide the necessary pre-clinical data to support testing of such strategies in clinical trials of patients with metastatic osteosarcoma.

## Results

### Longer duration PD-L1 mAb treatment provides no additional survival benefit to mice with metastatic osteosarcoma

**Figure 1:**
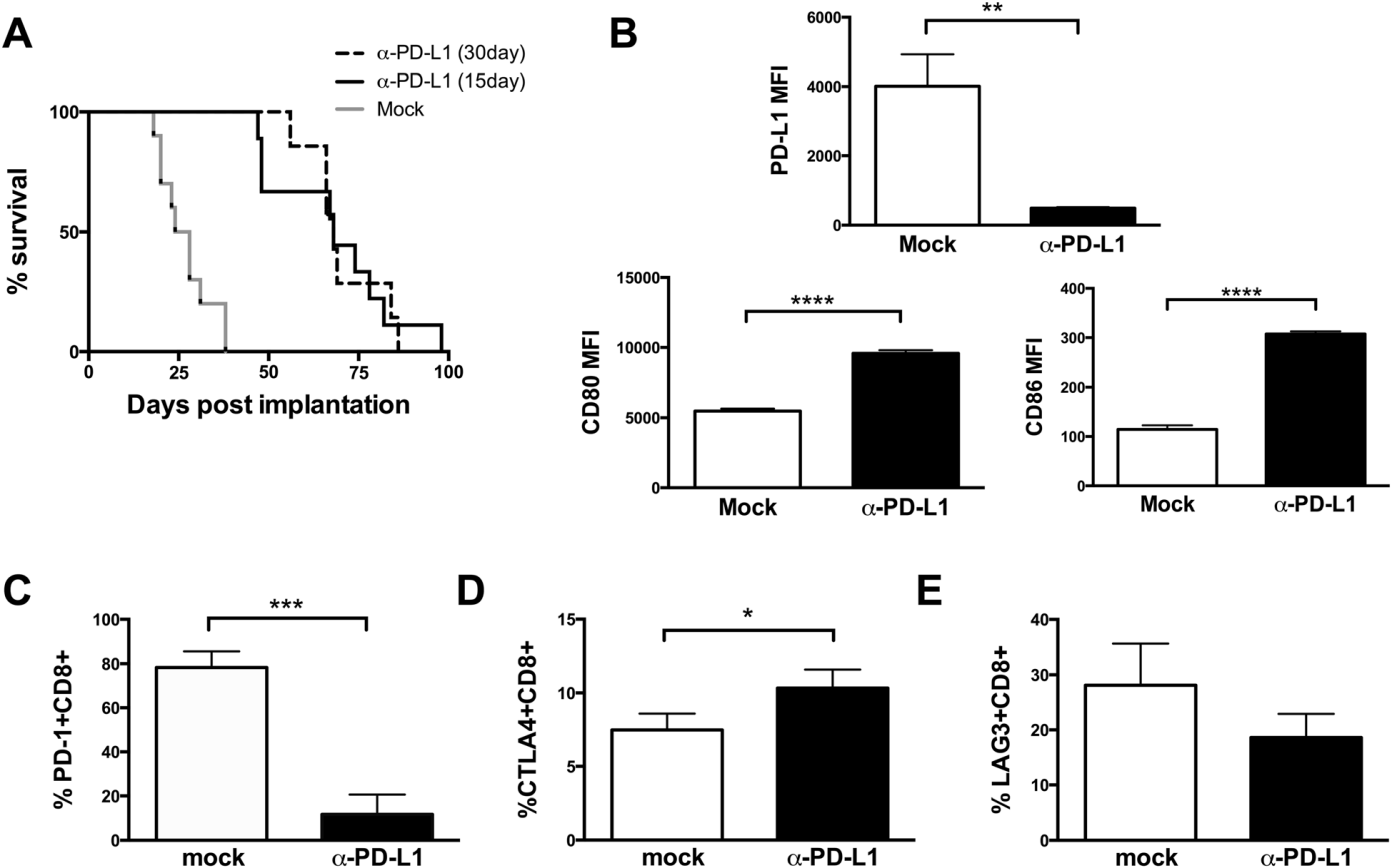
Higher dose and longer duration of α-PD-L1 mAb treatment does not improve survival, suggesting resistance to α-PD-L1 mAb treatment. Mice were injected with K7M2 cells, and treated for either 15 days (n = 10) or 30 days (n = 10) with α-PD-L1 mAb. Survival was not significantly different between the 15 day and 30 day treatment, p = 0.2942, and all mice succumbed to disease **(A)**. At time of necropsy, PD-L1, CD80, and CD86 expression was evaluated using Flow Jo. PD-L1 expression was significantly decreased in treated versus untreated mice, p = 0.0026. CD80 expression was significantly increased in treated versus untreated mice, p < 0.0001. CD86 expression was significantly increased in treated versus untreated mice, p < 0.0001 **(B)**. At time of necropsy, PD-1+, CTLA-4+, and LAG3+ CD8+ expression was evaluated using Flow Jo. PD-1 + CD8+ expression was significantly different in treated versus untreated mice, p = 0.0005 **(C)**. CTLA-4 + CD8+ expression was significantly different in treated versus untreated mice, p = 0.043 **(D)**. LAG3 + CD8+ expression was not significantly different between treated and untreated mice **(E)**.

Due to escape following PD-L1 mAb treatment, we wanted to test if PD-L1 mAb treated metastatic osteosarcoma is evading immune mediated killing, or if proliferation rate is outpacing immune mediated killing. To evaluate if longer α-PD-L1 mAb treatment could further improve T cell control of metastatic osteosarcoma disease, we doubled the dose and duration of α-PD-L1 mAb therapy. We observed no significant difference in the survival of metastatic osteosarcoma implanted mice when treated for 30 days vs 15 days; mice receiving α-PD-L1 mAb therapy perished from pulmonary metastases with a median survival of 68 days in both groups (Figure 1A). Therefore, we reasoned that metastatic osteosarcoma cells in these mice are becoming resistant to α-PD-L1 mAb therapy, and that this may be due, at least in part, to suppression of T cell responses by signaling via alternative immune inhibitory receptor pathways.

### Metastatic osteosarcoma treated with α-PD-L1 downregulate PD-L1 expression, but increase CD80/CD86 expression

To determine if T cell responses to osteosarcoma metastatses in α-PD-L1 mAb treated mice may have become tolerized through engagement of other inhibitory ligands, we evaluated expression of PD-L1, CD80, and CD86 on metastatic osteosarcoma cells, in both mock treated and α-PD-L1 mAb treated mice. PD-L1 expression on metastatic osteosarcoma was significantly decreased after α-PD-L1 mAb treatment suggesting that these cells are no longer co-opting the use of PD-1 to suppress T cell function (Figure 1B). Moreover, CD80 and CD86 expression on metastatic osteosarcoma was significantly increased after α-PD-L1 mAb treatment suggesting that tumor cells may be directly or indirectly using this pathway to suppress CTL-mediated killing (Figure 1B).

### Metastatic osteosarcoma reactive T cells from α-PD-L1 mAb treated mice decrease PD-1 but increase CTLA-4 expression

In order to determine if T cell responses to osteosarcoma metastases in α-PD-L1 mAb treated mice may have become tolerized through engagement of other inhibitory ligands, we evaluated expression of PD-1, CTLA-4, and LAG3 on tumor reactive T cells, as these inhibitory proteins have been shown to be critically important in other tumor tolerance settings [14-18]. PD-1 expression on CD8+ TILs was significantly decreased after α-PD-L1 mAb treatment suggesting that tumor cells are no longer co-opting the use of PD-1 to suppress T cell function (Figure 1C), either through downregulation of expression or via ineffective activation to initially upregulate this receptor. Conversely, tumor infiltrating CD8 T cells had higher levels of CTLA-4 expression in α-PD-L1 mAb blockade treated mice, suggesting that tumor cells may be directly or indirectly using this pathway to suppress CTL-mediated killing (Figure 1D). No statistical difference was observed in LAG3 expression after α-PD-L1 mAb treatment (Figure 1E).

### Metastatic osteosarcomas that develop in α-PD-L1 treated mice are resistant to additional PD-L1 blockade

**Figure 2:**
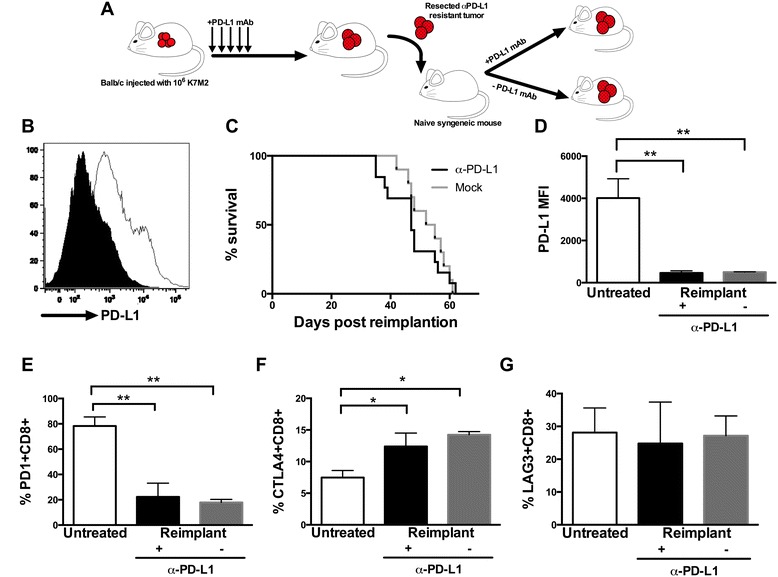
Naïve mice injected with *in vivo* treated α-PD-L1 mAb K7M2 cells are non-responsive to α-PD-L1 treatment, suggesting resistance to α-PD-L1 treatment. Naïve Balb/cJ mice were injected with *in vivo* α-PD-L1 mAb treated metastatic osteosarcoma, with decreased PD-L1 expression, **(B)** black histogram is PD-L1 expression prior to reimplantation in comparison to mock treated mice in white, and treated with α-PD-L1 mAb or mock **(A)**. No significant difference was seen between survival in treated (n = 10) and untreated (n = 10) mice injected with treated metastatic osteosarcoma **(C)**. PD-L1 expression on metastatic osteosarcoma from mice injected with *in vivo* α-PD-L1 mAb treated metastatic osteosarcoma compared to control K7M2 injected mice was significantly decreased, p = 0.0026 **(D)**. At time of necropsy, PD-1 + CTLA-4+, and LAG3+ CD8+ expression was evaluated using Flow Jo. PD-1 + CD8+ expression was significantly different in mice injected with *in vivo* α-PD-L1 mAb treated metastatic osteosarcoma compared to control K7M2 injected mice, p = 0.0017 **(E)**. CTLA-4 + CD8+ expression was significantly different in *in vivo* α-PD-L1 mAb treated metastatic osteosarcoma injected mice compared to control, p = 0.0231 **(F)**. LAG3 + CD8+ expression was not significantly different between these two groups **(G)**.

We also observed lower expression of PD-L1 on K7M2 metastatic osteosarcoma cells from α-PD-L1 mAb-treated mice compared to tumor cells from mock-treated mice (Figure 2B). This further suggested that these cells may be using alternative strategies to evade immune clearance. To evaluate if down-regulation of PD-L1 by K7M2 metastatic osteosarcoma cells after α-PD-L1 mAb treatment resulted in resistance to further α-PD-L1 mAb treatment, versus indirect inhibition by other tumor resident cells, metastastatic osteosarcoma cells isolated from α-PD-L1 mAb treated mice were re-implanted into naïve recipient mice that were then subsequently treated with α-PD-L1 mAb or received mock injections (Figure 2A). We reasoned that if PD-L1 resistance was due to indirect effects via tumor-resident inhibitory or regulatory cells, the tumors would become sensitive to PD-L1 blockade after re-implantation and re-establishment of the tumor. Strikingly, mice injected with osteosarcoma cells from α-PD-L1 mAb treated mice and treated with additional α-PD-L1 mAb showed no difference in survival compared to mock treated mice (Figure 2C), suggesting that K7M2 cells were directly inhibiting TILs by alternative pathways. An alternative explanation is that PD-L1 mAb treatment may be altering the microenvironment towards a T cell suppressive state, however increases in CD80/86 expression on the tumor cells after PD-L1 treatment suggest direct K7M2 TIL inhibition. Tumor cells from α-PD-L1 mAb treated mice appeared to have overall slower growth kinetics in vitro, compared to the parental tumor cells, resulting in an overall longer mean survival of ~50 days versus only ~25 days after reimplantation of tumors from untreated mice, which is to be expected as PD-L1 has been shown to speed up tumor cell growth kinetics and provide anti-apoptotic signals [19,20] (Additional file 1: Figure S1). Tumor cells from α-PD-L1 mAb treated mice retained low expression levels of PD-L1, compared to tumor cells from untreated mice, whether or not additional α-PD-L1 mAb was administered (Figure 2D) and CD8+ TILs isolated from PD-L1 mAb resistant tumors continued to exhibit decreased expression of PD-1 but elevated expression of CTLA-4, while LAG3 expression remained unchanged (Figure 2E-G). Taken together,these data support the idea that immunoselection of K7M2 metastatic osteosarcoma cells that eventually cause lethal disease in α-PD-L1 mAb treated mice evokes an adaptive resistance mechanism in the microenvironment, and instead the tumor or tumor microenvironment may be using CTLA-4 ligation as an alternative pathway to escape immune destruction.

### Combination of PD-L1 mAb blockade with CTLA-4 mAb blockade can result in complete control of metastatic osteosarcoma tumors in a subset of mice

**Figure 3:**
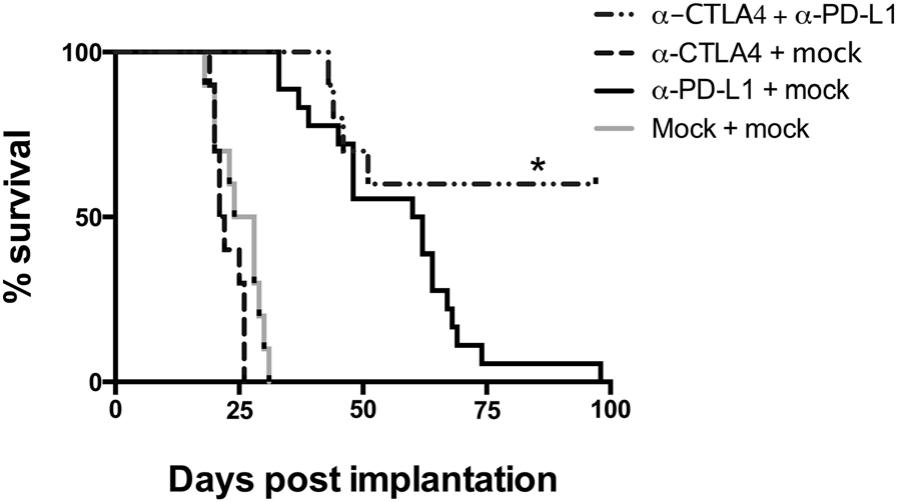
Dual α-CTLA-4/α-PD-L1 mAb treatment can completely eradicate metastatic osteosarcoma. Survival of combinational treatment of implantable metastatic osteosarcoma with α-CTLA-4 and α-PD-L1 mAb was significantly higher than α-PD-L1 mAb treatment alone, p = 0.0177. Additionally, survival of combination treatment α-CTLA-4/α-PD-L1 mAb compared to α-CTLA-4 mAb treatment alone was significantly different with a p value of p < 0.0001. α-CTLA-4 mAb alone was not significantly different than mock treated mice. N = 10 for all treatment groups.

Due to the increased CTLA-4 expression observed on TILs after selection of α-PD-L1 resistant tumors, and increased CD80/86 expression on remaining tumor cells, we hypothesized that blockade of both CTLA-4 and PD-L1 might lead to better tumor control. Combination of α-CTLA-4 and α-PD-L1 mAb treatment resulted in complete control of metastatic osteosarcoma tumors with long-term disease-free survival in roughly 60% of α-CTLA-4 + α-PD-L1 mAb treated mice. This was in comparison to 0% long-term survival of mice treated with α-PD-L1 alone (p = 0.0177, Figure 3). Moreover, the combination of these antibody blockade strategies appeared to have a synergistic effect as α-CTLA-4 mAb treatment alone showed no benefit in slowing the progression of metastatic osteosarcoma compared to untreated mice. Additionally, tumor-specific TIL function is enhanced at day 25 in dual treated mice in comparison to CTLA4 mAb alone, PD-L1 mAb alone, or mock treated mice (Additional file 2: Figure S2).

### Dual PD-L1/CTLA-4 treatment and control of metastatic osteosarcoma leads to protective immunity against future tumor inoculation

**Figure 4:**
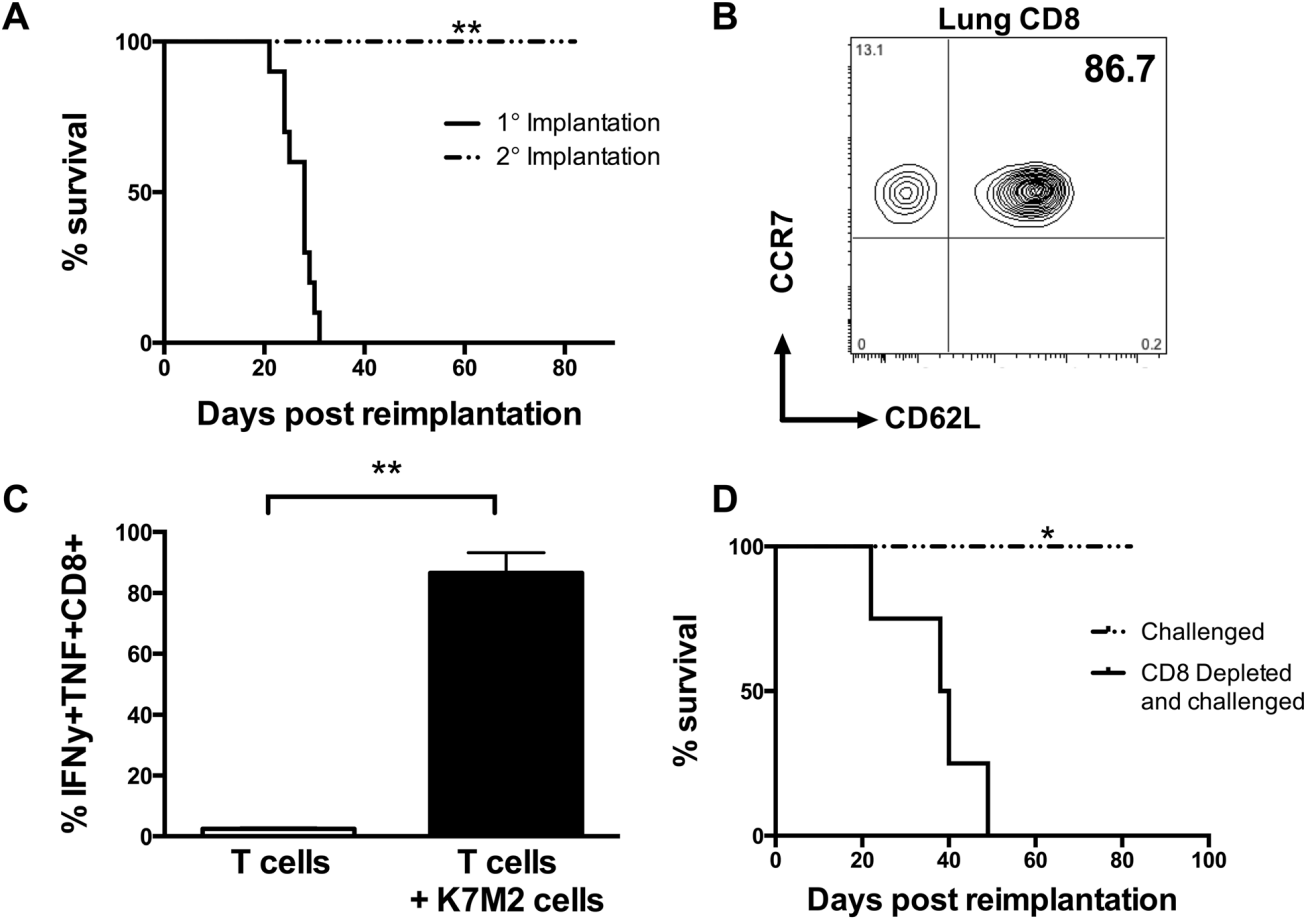
Dual α-CTLA-4/α-PD-L1 mAb treatment can elicit protective immunity towards metastatic osteosarcoma. To evaluate protective immune responses to tumor, cured α-CTLA-4/α-PD-L1 mAb mice (n = 12) were challenged with 10^6^ K7M2 cells at day 100. Cured α-CTLA-4/α-PD-L1 mAb treated mice cleared disease and significantly survived longer than age-matched control mice, p = 0.0049 **(A)**. Lung tissue was evaluated for the presence of memory CD8 cells, and approximately 87% of the tumor-reactive memory T cells were CCR7 + CD62L+ **(B)**. These CCR7 + CD62L + CD8+ cells were specific towards K7M2 cells used to generate the tumor, and able to produce IFNy and TNF in response to re-exposure, p=0.002 **(C)**. To evaluate if protective immune responses to tumor were in fact due to memory CD8 T cells. Cured α-CTLA-4/α-PD-L1 mice (n = 6) were depleted of CD8 T cells at day 80, and challenged with 10^6^ K7M2 cells after depletion. Mice depleted of CD8 T cells prior to challenge all succumbed to metastatic osteosarcoma in the lung tissue, in comparison to 100% survival (n = 6) in cured immune competent mice challenged. A log-rank test gives a p = 0.0177 **(D)**.

In order to determine if eradication of tumors in combination α-CTLA-4 and α-PD-L1 mAb treated mice resulted in sterilizing anti-tumor immunity, we next evaluated if the α-CTLA-4/α-PD-L1 mAb treated mice were protected against subsequent tumor inoculation. Mice previously treated with α-CTLA-4 and α-PD-L1 mAb and that had previously controlled metastatic osteosarcoma were challenged with 10^6^ K7M2 cells at 100 days post-initial inoculation, a time when neither therapeutic antibody should be present in these mice. Mice that had controlled metastatic osteosarcoma were selected via no detectable tumors (4 of surviving mice at day 100 were euthanized, Additional file 3: Figure S3), and no physical signs of disease at day 100. Strikingly, α-CTLA-4/α-PD-L1 mAb treated mice that had controlled initial K7M2 tumors, were completely immune to challenge with additional K7M2 cells, and remained tumor free for an additional 80 days when they were euthanized to evaluate immune memory (Figure 4A). At the time of necropsy (180 days post initial tumor inoculation), these tumor–immune mice also had no visible pulmonary metastases (Additional file 3: Figure S3).

We next asked whether immunity to metastatic osteosarcoma in combination α-CTLA-4/α-PD-L1 mAb treated mice that controlled initial K7M2 tumors correlated with retention or improved T cell function. Perfused lung tissue from healthy mice had few CD8 cells present. In our previously published work, mock-treated mice implanted with K7M2 cells had many more TILs, but these were largely unresponsive to antigen-stimulation with 86.07 ± 0.8253% of TILs expressing PD-1 [6]. In contrast, in dual treated α-CTLA-4/α-PD-L1 mAb mice a large number of TILs were also observed in α-CTLA-4/α-PD-L1 mAb treated mice. However the vast majority (86.63 ± 6.661%) of the CD8 cells present in the lungs of combination treated mice were able to produce both IFNy and TNF in response to stimulation with parental K7M2 cells *in vitro* (Figure 4C). This difference in function between treated and control mice correlated with a central memory phenotype (CD62L + CCR7+) (Figure 4B) as 85% of TILs from α-CTLA-4/α-PD-L1 mAb treated mice were CCR7 + CD62L+ while most cells from mock-treated mice were low for these surface markers. Thus, the presence of high numbers of central-memory phenotype and cytokine positive CD8 T cells in the lung tissue from dual α-CTLA-4/α-PD-L1 mAb treated mice likely mediate protective immunity to further K7M2 re-exposure.

In order to directly test whether the central-memory phenotype CCR7 + CD62L + CD8+ TILs in tumor-immune mice mediated resistance to subsequent tumor inoculation, we depleted CD8 T cells at day 80 after primary tumor control in α-CTLA-4/α-PD-L1 mAb treated mice with 2.43 hybidoma purified antibodies, followed by re-challenge with additional K7M2 tumor cells. All mice that were CD8 depleted prior to K7M2 re-challenge succumbed to metastatic osteosarcoma, with a median survival of ~40 days. This was in comparison to control α-CTLA-4/α-PD-L1 mAb treated tumor-immune mice that were re-challenged with K7M2 cells without CD8 depletion and showed no evidence of lung metastases or disease (Figure 4D, Additional file 3: Figure S3).

### Combination of PD-L1 mAb blockade with chemotherapy shows no improvement in control of metastatic osteosarcoma tumors

**Figure 5:**
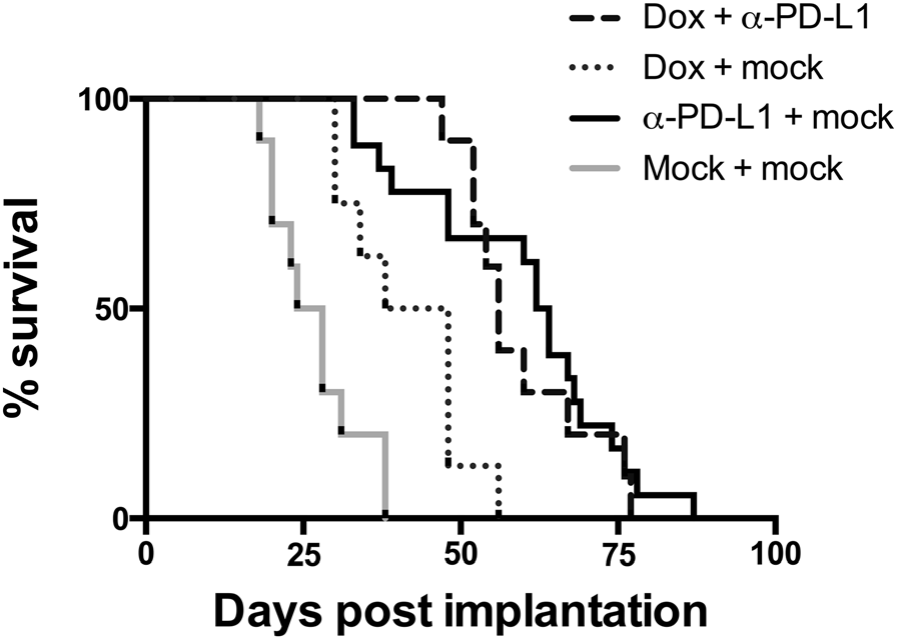
α-PD-L1 mAb treatment coupled with doxorubicin treatment does not enhance survival over α-PD-L1 mAb treatment alone. Survival of combinational treatment of metastatic osteosarcoma with doxorubicin and α-PD-L1 mAb was significantly higher than chemotherapy treated alone group, p = 0.0026, although no significant difference in survival was seen between combinational α-PD-L1 mAb and doxorubicin treatment and α-PD-L1 mAb alone. N = 10 for all treatment groups.

We next tested whether chemotherapy can enhance the curative potential of PD-L1 blockade during metastatic osteosarcoma progression in the K7M2 model, primarily to test if the current standard of care in combination with PD-L1 mAb can also elicit synergistic protection versus anti-tumor effects. We hypothesized that combination with α-PD-L1 mAb blockade may result in increased presentation of tumor antigens to T cells and higher magnitude responses that may, therefore, avoid the observed escape of tumors from this treatment, as well as slow the overall growth of tumors to facilitate immune control. CD4 and CD8 T cells are resistant to doxorubicin treatment with similar stimulation between mock and doxorubicin treated mice, however CD4 T cells can exhibit enhanced proliferation due to increased expression of CD40 ligand and 4-1BB when treated with doxorubicin both *in vitro* and *in vivo* [21]*.* While doxorubicin chemotherapy alone was effective in treating metastatic osteosarcoma, p-value = 0.0048, we observed no additional improvement in the survival of mice treated with doxorubicin + α-PD-L1 mAb compared to α-PD-L1 mAb treatment alone (Figure 5). Additionally, we see similar trends in decreases in PD-1 and increases in CTLA4 expression on CD8 T cells, and decreases in Mean Fluorescent Intensity of PD-L1, during PD-L1 mAb treatment regardless of doxorubicin treatment (Additional file 4: Figure S4). Thus, combination of chemotherapy with immunotherapy approaches do not appear to have additional beneficial effects on tumor control and mice eventually succumb to disease similar to progression during α-PD-L1 mAb treatment alone.

## Discussion and conclusions

The prognosis for patients with metastatic osteosarcoma remains dismal, in part, due to tumor resistance to conventional chemotherapy or radiation treatments. We have recently shown that α-PD-L1 blockade can improve survival and slow metastatic osteosarcoma progression by partially restoring T cell function towards this tumor. However, mice receiving immunotherapy with α-PD-L1 mAb eventually succumb to pulmonary metastatic disease. Here we show that metastatic osteosarcoma tumors from mice treated with α-PD-L1 mAb down-regulate expression of PD-L1, and become resistant to further blockade treatment. Tumor resistance to this immunotherapy appeared to be direct inhibition of TILs via ligation of other inhibitory receptors, and correlated with an increase in the expression of CTLA-4 on TILs. Combination blockade with α-CTLA-4 and α-PD-L1 mAb resulted in complete control of metastatic osteosarcoma and sterilizing immunity to further tumors. This effect appeared to be synergistic as α-CTLA-4 blockade alone had no impact on tumor control, and α-PD-L1 blockade coupled with the chemotherapy to slow tumor growth had no impact on survival over α-PD-L1 blockade alone. Higher doses of this CTLA4 mAb clone may be needed to elicit significant survival benefits in CTLA4 mAb treated mice alone, however, at this low dose we were still able to see a synergistic effect when coupled with PD-L1 mAb treatment. This may be of some advantage when treating pediatric patients, as high dose CTLA4 mAb treatment can lead to significant secondary side effects. Thus, combinational α-PD-L1/α-CTLA-4 blockade is an attractive combinational therapy to use in humans to enhance T cell mediated rejection of metastatic osteosarcoma with the ultimate goal of improving patient prognoses while avoiding tumor escape.

This paper, to our knowledge, provides the first direct evidence of immune escape by metastatic osteosarcoma in response to α-PD-L1 blockade immunotherapy. Blockade treatment reinvigorates T cell function, which can ultimately drive immune-mediated tumor cell selection if complete eradication does not occur. Accumulating evidence suggests that although the immune system identifies and eliminates pre-cancerous and cancerous cells, tumors still develop in immune competent individuals due to ineffective elimination, resulting in an equilibrium phase of tumor development [22-25]. During such an equilibrium phase, selective pressure by the immune system on the tumor, with genomic instability of the tumor cells, leads to additional heterogeneity, with the potential for shifts in antigen presentation, reduction of costimulatory proteins, or increases in inhibitory proteins, and eventual tumor escape from immune control. In particular, tumor cells up-regulate ligands such as PD-L1 to exhaust T cell responses [26] and limit immune-mediated killing [27-30]. Alternative immune checkpoint controls (CTLA-4, PD-1, LAG-3, TIM-3) on T cells provide tumors with several options for avoiding tumor-reactive immune responses [31-36,28]. We propose that use of α-PD-L1 blockade alone in treating metastatic osteosarcoma, partially reinvigorates T cell function resulting in slower tumor growth, but allows for tumor escape by signaling via alternative immune inhibitory receptors and eventual selection of resistant tumors that cause lethal disease.

We have observed an upregulation of CTLA-4 expression on TILs, following α-PD-L1 treatment alone, as an alternative mechanism by which tumor cells can escape T cell responses. Certainly, clinical approaches and experimental systems using this antibody support our conclusions as similar results have been previously shown in a B16 mouse model of melanoma, in which a two-fold increase in the percentage of CTLA-4 expressing cells after treatment with anti-PD-1 mAb was observed [8]. A similar effect has also been observed in patients treated with PD-1 blockade, in which increased CTLA-4 expression on TILs has been noted (*personal communication* between J. Weber and M. Sznol [37]. Thus, it is likely that this is a common mechanism by which tumor cells can co-opt sequential inhibition of different signaling pathways in T cells leading to disease progression.

The dual α-CTLA-4/α-PD-L1 treatment in our metastatic osteosarcoma mouse model appears to be synergistic, as we observed no benefit after provision of α-CTLA-4 mAb alone. These synergistic survival effects in mice treated with α-CTLA-4/α-PD-L1 mAb may be mediated by TILs promoting tumor clearance via blockade of non-overlapping pathways, which may lead to greater restoration of T cell function, or sequential blockade of these pathways resulting from the dynamic expression of PD-1 and CTLA-4 during T cell responses. Overall, greater TIL function after stimulation with K7M2 cells is seen in dual blockade treated mice at day 25 post treatment, in comparison to PD-L1 mAb alone, CTLA4 mAb alone, or mock treatment (Additional file 2: Figure S2). Our data suggests that CTLA-4 is upregulated on CD8+ TILs only after α-PD-L1 blockade, favoring the latter mechanism. The inhibitory pathways of PD-1 and CTLA-4 appear to be non-redundant with distinct mechanisms in maintenance of peripheral tolerance, making the combination of the two of particular interest in conferring synergistic tumor effects mediated by CD8+ TILs [38].

An alternative explanation for the observed synergy during combinational α-CTLA-4/α-PD-L1 blockade of metastatic osteosarcoma is that tumor-resident regulatory or suppressor cells may be limiting the function of TILs, and each antibody may be targeting pathways in different cell types. Certainly, multiple studies have shown that CTLA-4 blockade can deplete tissue-resident regulatory T cells, and this in turn may indirectly affect CD8+ TIL function, conferring control by alleviating immune suppression at the tumor site [38,8]. However, the CTLA4 mAb clone UC10-4F10-11, and dose used, suggest that this is not decreased regulatory T cell mediated effects on function. Alternatively, α-PD-L1 blockade may alter the tumor microenvironment, in a manner favoring T cell suppression by pathways other than PD-1, and therefore this may not be direct K7M2 cell inhibition leading to resistance. However, our results showing continued PD-L1 mAb resistance after re-establishment of tumors in naïve mice suggest that such accessory cells are likely not the primary mechanism by which TILs are limited in their ability to mediate tumor killing. Rather, we propose that K7M2 cells are directly inhibiting TILs leading to T cell exhaustion, and that combination α-CTLA-4/α-PD-L1 blockade is preventing or reversing this process, as heightened CD80/CD86 expression is seen on the tumor cells post α-PD-L1 blockade, mirroring the increased CTLA4 expression on the tumor-reactive TILs following treatment.

The Children’s Oncology Group (COG) has recently launched a Phase I/II trial in pediatric patients with relapsed/refractory solid tumors of nivolumab (anti-PD1 inhibitor) either alone or in combination with ipilimumab (anti-CTLA-4 antibody). In the phase II portion of the trial, osteosarcoma will be one of the expansion cohorts to assess efficacy of these agents. Based on the results from this paper, combinational immunotherapy coupling α-CTLA-4 and α-PD-L1 mAb treatment may have the best chance of increasing survival in metastatic osteosarcoma patients. One of the major challenges of immune therapies in pediatric patients is the significant toxicity profile of these agents seen in all types of patients, and it would be imperative to find the most tolerable yet effective combination regimen for these agents in pediatric patients [39]. The National Cancer Institute recently approved a Phase I study of Ipilimumab in children and adolescents with treatment-resistant cancers, however no information has been published regarding this phase I trial (NCT01445379). The COG trial would be instrumental in providing the safety and dosing data for pediatric patients. Results of our study and other similar studies in preclinical models have generated a tremendous amount of excitement regarding the huge potential for immunotherapy of cancer and will provide further incentive for the rational development of more efficacious and safer targets to improve resistance to metastatic tumors.

## Methods

### Antibodies and cell lines

Fluorochrome-conjugated anti-mouse monoclonal antibodies (Abs) specific for CD8α, CD274, CD279, CTLA-4, CD80, and CD86 were purchased from eBiosciences (San Diego, CA). The anti-PD-L1 monoclonal antibody (clone 10F.9G2) used for *in vivo* blockade experiments was purchased from BioXCell (West Lebanon, NH). The anti-CTLA-4 monoclonal antibody was purified from the UC10-4F10-11 hybridoma, (ATCC, Manassas, VA). K7M2 osteosarcoma cells were purchased from ATCC, and screened by IDEXX Laboratories (Columbus, MO).

### Mice and generation of tumors

3–4 week-old Balb/cJ mice were purchased from the Jackson Laboratories (Bar Harbor, ME) and maintained under specific pathogen-free conditions in Arizona State University Biodesign Institute animal facilities. All experiments were approved by the Arizona State University Institutional Animal Care and Use Committee, and conducted under appropriate supervision. To establish metastatic osteosarcoma tumors in mice, 10^6^ K7M2 cells were injected via the lateral tail vein in 100 μL of Hanks Balanced Salt Solution. Both weight loss and a clinical scoring system were used to monitor for the development of metastatic lung disease, with a mean time to diagnosis of 24 days from injection of cells. Mice were ranked from 0 (normal) to 3 (abnormal) in mentation/appearance, respiration, ambulation, and for the occurrence of tremors/convulsions. Mice were euthanized for analysis by CO_2_ asphyxiation when weight loss was >10% and/or physical symptoms (a cumulative score > 6 or a score of 3 in any individual category) were observed. For significance, all treatment groups consisted of n = 10 or more.

### Lung preparation

Mice with metastatic pulmonary disease were anesthetized with a mouse ketamine cocktail administered IP using the dose of 42mg/kg ketamine, 4.8 mg/kg xylazine, and 0.6 mg/kg acepromazine followed by lung perfusion with ice-cold PBS to remove peripheral blood mononuclear cells (PBMC). Mice were then euthanized as described above. Lungs were collected in RPMI media and Metastatic osteosarcoma-infiltrating cells were isolated from collagenase-treated lung tissue by centrifugation over a 30/90% Percoll gradient (Sigma-Aldrich, St. Louis, MO) and collection of interface cells before antibody staining of cell populations on an LSRFortessa II flow cytometer (BD Biosciences, San Jose, CA) [40]. Flow cytometry data were analyzed with FlowJo8.8 (Tree Star Inc., Ashalnd, OR) and graphs generated with Prism5 software (GraphPad Software, La Jolla, CA). Statistical significance reported * p < 0.05, ** p < 0.01, *** p < 0.001, and **** p < 0.0001.

### Intracellular cytokine staining

Lymphocytes were cultured alone or stimulated with K7M2 cells. GolgiStop (BD Biosciences) was added at 1 hour to inhibit export of cytokines and after a further 5 hours of incubation, cells were stained for extracellular proteins [41]. Permeabilization and intracellular staining for cytokines was done according to manufacturer’s instructions using the Cytofix/Cytoperm kit (BD/Pharmingen).

### Cytotoxicity ELISA

Lymphocytes were isolated from lung tissue, and cultured alone or with K7M2 cells. LDH Elisa was performed using CytoTox 96 Non-Radioactive Cytotoxicity Assay (Promega, Madison, WI) and absorbance was recorded at 490nm.

### *In vivo* PD-L1 antibody blockade

Mice inoculated with K7M2 cells as described above were administered 200 μg PD-L1 antibody (10F.9G2) in PBS or mock PBS control intraperitoneally every three days, starting one day after tumor inoculation [42].

### *In vivo* CTLA-4 antibody blockade

Supernatant from UC10-4F10-11 hybridoma cells [43] was precipitated in saturated ammonium sulfate to 45% (v/v) overnight at 4°C and dialyzed against PBS for 24 hrs. The concentration of dialyzed antibody was determined by UV spectrophotometer analysis using a Nanodrop (Thermo Scientific). Mice inoculated with K7M2 cells as described above were administered 100 μg CTLA-4 antibody in PBS or mock PBS control intraperitoneally every three days, starting one day after tumor inoculation [44,45].

### *In vivo* doxorubicin treatment

Doxorubicin hydrochloride (Sigma-Aldrich), was injected at 2mg/kg into the lateral tail vein of K7M2 inoculated mice at +7 days from tumor cell injection [46].

### Reimplantation of PD-L1 treated metastatic osteosarcoma

Mice inoculated with K7M2 cells were euthanized after presenteding with clinical symptoms. Metastatic osteosarcoma lung tissue was processed as described above, without Percoll gradient separation. After cell counting and staining to determine the percentage of osteosarcoma cells, 10^6^ tumor cells were re-implanted into naïve 3–4 week old Balb/cJ mice.

### PD-L1 knock-down in K7M2 cells

293FT cells were purchased from ATCC, and were plated at a low confluency on 10cm dishes. The ViraPower Lentiviral Expression System, purchased from Life Technologies (Carlsbad, CA), was incubated with the CD274-set siRNA/shRNA/RNAi Lentivector, purchased from Applied Biological Materials (Richmond, BC), to transfect 293FT cells and incubated overnight at 37 degrees prior to harvesting the virus. Virus was used to transduce K7M2 cells by incubating for 48 hrs at 37 degrees. Once the transduced K7M2 cells were confluent, cells were stained with anti-PD-L1 and Propidium Iodide followed by analysis of cell populations on an LSRFortessa flow cytometer. Flow cytometry data were analyzed with FlowJo8.8 and ModFitLT, and graphs generated with Prism5 software. Paired T test was used to generate statistical significance.

## Additional files

Additional file 1: Figure S1. PD-L1 expression enhances K7M2 cell proliferation and promotes anti-apoptotic signals. Flow cytometry plots gating on K7M2 PD-L1 positive cells versus K7M2 PD-L1 negative cells from flask treated with lentiviral vector containing PD-L1 shRNA, propidium iodide stain reveals number of cells in G1, G2, and S phase (A). Graphical representation of the different percentage of cells in G1 versus G2 and S between K7M2 PD-L1 positive cells versus K7M2 PD-L1 negative cells. PD-L1 expression significantly decreased the number of cells in G1, p < 0.01. PD-L1 expression significantly increased the number of cells in G2 and S, p < 0.01 (B). Percentage of apoptosis reveals that PD-L1 expression on K7M2 cells decreases amount of apoptosis significantly, p < 0.01 (C). Additional file 2: Figure S2. Dual blockade treated tumor-specific TILs are more functional at day 25 in comparison of PD-L1 mAb alone, CTLA4 mAb alone, or mock treated mice. TILs isolated from mice of varying treatments were cultured alone, or with K7M2 cells used to generate the tumor. IFNy, TNF, and IL-2 function was evaluated (A, B, and C). Mice treated with CTLA4 and PD-L1 mAbs had significantly higher IFNy production in comparison to mice treated with either PD-L1 mAb alone, CTLA4 mAb alone, or mock treated, p < 0.0001 (A). Mice treated with CTLA4 and PD-L1 mAbs had significantly higher TNF production in comparison to mice treated with either PD-L1 mAb alone p < 0.05, CTLA4 mAb alone p < 0.01, or mock treated p < 0.001 (B). Mice treated with CTLA4 and PD-L1 mAbs had significantly higher IL-2 production in comparison to mice treated with either PD-L1 mAb alone p < 0.01, CTLA4 alone p < 0.001, or mock treated mice p < 0.001 (C). LDH ELISA confirming increased TIL function in dual treated mice with a significant increase in % cytotoxicity between PD-L1 mAb alone p < 0.01, CTLA4 alone p < 0.0001, or mock treated p < 0.0001 (D). Additional file 3: Figure S3. Pictures of mouse lung tissue with varying treatments. Healthy lung tissue (A). K7M2 metastatic disease with no treatment (B). K7M2 metastatic disease at time of death in PD-L1 mAb blockade treated mice (C). Mice injected with K7M2 cells and treated with both PD-L1 and CTLA4 mAbs, and osteosarcoma controlled. No physical signs of disease, euthanized at day 100 (D). Osteosarcoma immune mice, challenged at day 100, euthanized with no physical signs of disease at day 180 (E). Additional file 4: Figure S4. TIL phenotype in dual treated α-PD-L1 mAb and doxorubicin treatment. N = 10 for all treatment groups. At time of necropsy, PD-1+ and CTLA-4+ CD8+ expression was evaluated using Flow Jo. PD-1 + CD8+ expression was significantly different in dual doxorubicin and PD-L1 mAb treatment versus doxorubicin alone mice, p < 0.001 (A). CTLA-4 + CD8+ expression was significantly different in PD-L1 mAb treatment versus doxorubicin alone mice, p < 0.01 (B). PD-L1 xpression was significantly decreased in dual doxorubicin and PD-L1 mAb treatment versus doxorubicin alone mice, p < 0.01.

## Abbreviations

PD-1: Programmed death receptor-1
CTL: Cytotoxic T lymphocytes
PD-L1: Programmed death receptor-1 ligand
CTLA4: Cytotoxic T-lymphocyte-associated protein 4
LAG3: Lymphocyte-activation gene 3
TIL: Tumor infiltrating lymphocyte
mAb: Monoclonal antibody
TIM-3: T cell immunoglobulin mucin-3
COG: Children’s oncology Group
