# Supplementary figures and images for: Combination immunotherapy with α-CTLA-4 and α-PD-L1 antibody blockade prevents immune escape and leads to complete control of metastatic osteosarcoma
Source: J Immunother Cancer. 2015 May 19;3:21. doi: 10.1186/s40425-015-0067-z (PMC4437699; doi:10.1186/s40425-015-0067-z)

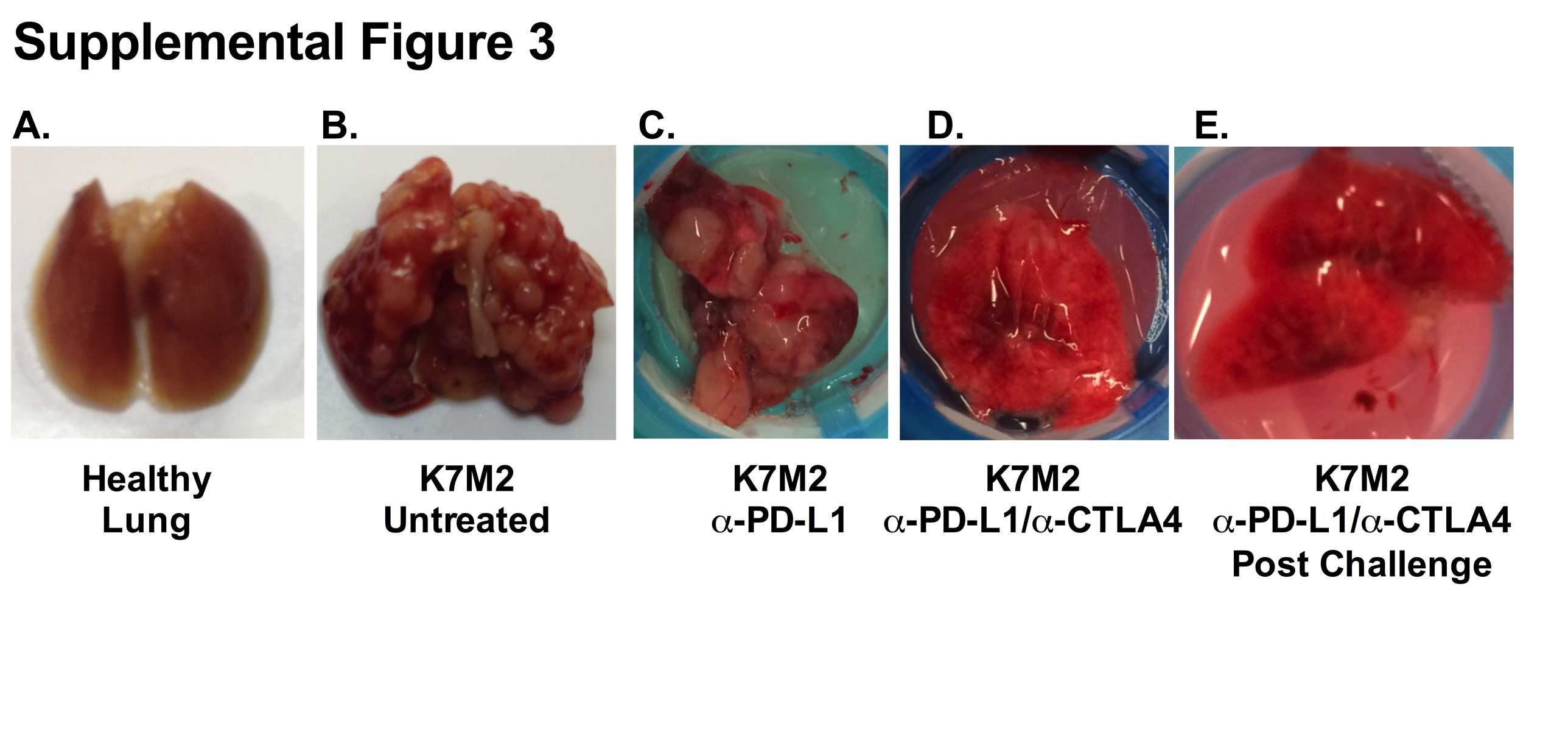

Supplement: Additional file 3: Figure S3. — Pictures of mouse lung tissue with varying treatments. Healthy lung tissue (A). K7M2 metastatic disease with no treatment (B). K7M2 metastatic disease at time of death in PD-L1 mAb blockade treated mice (C). Mice injected with K7M2 cells and treated with both PD-L1 and CTLA4 mAbs, and osteosarcoma controlled. No physical signs of disease, euthanized at day 100 (D). Osteosarcoma immune mice, challenged at day 100, euthanized with no physical signs of disease at day 180 (E). [file 40425_2015_67_MOESM3_ESM.tiff]
